# Severity, Pathogenicity and Transmissibility of Delta and Lambda Variants of SARS-CoV-2, Toxicity of Spike Protein and Possibilities for Future Prevention of COVID-19

**DOI:** 10.3390/microorganisms9102167

**Published:** 2021-10-18

**Authors:** Mehrnoosh Moghaddar, Ramtin Radman, Ian Macreadie

**Affiliations:** 1 School of Science, RMIT University, Bundoora, VIC 3083, Australia; mehrnoosh55m@gmail.com (M.M.); ramtinradman@gmail.com (R.R.); 2 School of Health and Medicine, Monash University, Clayton, VIC 3800, Australia

**Keywords:** SARS-CoV2, Delta variant, Lambda variant, spike protein toxicity, ACE2, furin, N protein

## Abstract

The World Health Organization reports that SARS-CoV-2 has infected over 220 million people and claimed over 4.7 million lives globally. While there are new effective vaccines, the differences in behavior of variants are causing challenges in vaccine development or treatment. Here, we discuss Delta, a variant of concern, and Lambda, a variant of interest. They demonstrate high infectivity and are less responsive to the immune response in vaccinated individuals. In this review, we briefly summarize the reason for infectivity and the severity of the novel variants. Delta and Lambda variants exhibit more changes in NSPs proteins and the S protein, compared to the original Wuhan strain. Lambda also has numerous amino acid substitutions in NSPs and S proteins, plus a deletion in the NTD of S protein, leading to partial escape from neutralizing antibodies (NAbs) in vaccinated individuals. We discuss the role of furin protease and the ACE2 receptor in virus infection, hotspot mutations in the S protein, the toxicity of the S protein and the increased pathogenicity of Delta and Lambda variants. We discuss future therapeutic strategies, including those based on high stability of epitopes, conservation of the N protein and the novel intracellular antibody receptor, tripartite-motif protein 21 (TRIM21) recognized by antibodies against the N protein.

## 1. Introduction

SARS-CoV-2, the cause of COVID-19, is a pathogenic member of the *Coronavirinea* subfamily and Betacoronavirus genera, which consists of an enveloped, unsegmented, single-stranded, positive sense RNA of ~29.9 kb [1,2]. Patients with COVID-19 demonstrate symptoms ranging from common cold-like symptoms to severe symptoms in multiple organs and, in some cases, death [3]. According to the serotype and genomic classification, there are four *Coronavirinae* subfamily variants of concern (VOC) reported by the World Health Organization (WHO, Alpha (B.1.1.7), Beta (B.1.351), Gamma (P.1) and Delta (B.1.617.2). Currently, two further variants, denoted as variants of interest (VOI), include Lambda (C.37) and Mu (B.1.621) [2,4]. Due to the high mutability in SARS-CoV-2, it is predicted that more contagious variants that evade current vaccines may appear in the future [5]. The high transmissibility of Delta and Lambda variants has raised the need to determine the reason for the infectivity of these variants. Since the Delta variant first emerged in India in June 2020, the global incidence of the Delta variant has made it a VOC [6]. The Delta variant is 60% more infectious than the original Wuhan SARS-CoV-2 strain [7]. Similarly, Lambda, a VOI, was first reported in Peru in December, 2020 and currently, its diverse mutations and increased frequency in over 29 countries has raised more attention [8]. Various studies have been conducted to identify the structure of the proteins of the virus to determine the reason for the higher infectiousness of the Delta and Lambda variants in comparison with the previous lineages. Among the proteins of the virus, spike protein (S) plays a crucial role in pathogenicity by attaching to the host cell. This property of the spike protein has prompted attention for its use in vaccine development. However, the S protein is highly variable, resulting in challenges for its ability to be an on-going therapeutic target. This paper reviews the reason for the high infectivity and vaccine evasion of Delta and Lambda, focusing on the role of the S protein in increasing the infectivity, considering other proteins of the virus as potentially better therapeutic targets.

## 2. Genome Structure of SARS-CoV-2 Virus

**Figure 1:**
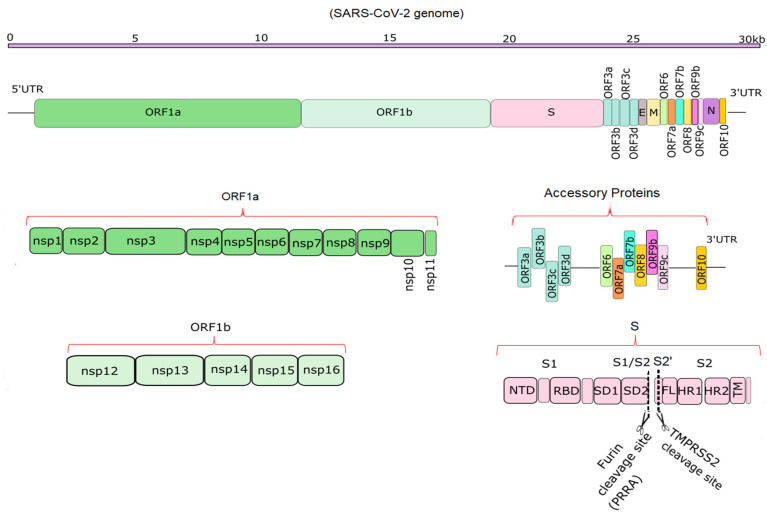
Schematic diagrams of the SARS-CoV-2 genome and its translation products. The top part shows the ~29.9 kb genome [1]. The second portion shows the overall position of structural, non-structural and accessory proteins in 5′ to 3′ order [3,21]. The bottom half depicts the non-structural proteins encoded by ORF1a (NSP1-11) and ORF1b (NSP12-16), 11 accessory proteins encoded by ORF3-ORF10, and the structure of S, which has S1 and S2 domains [21]. Furin and TMPRSS2 cleavage sites (PRRA and S2′ sites) are depicted in the S protein [22].

Compared to SARS-CoV and MERS-CoV, the SARS-CoV-2 genome has 82% sequence identity overall, and over 90% sequence identity for structural proteins and essential enzymes [9]. According to the NCBI annotation, (NC_045512), SARS-CoV-2 has 14 functional open reading frames (ORFs), which encode up to 31 proteins, shown schematically in Figure 1 [3,10]. The first ORFs, ORF1a and ORF1b, are the longest ORFs and occupy more than two thirds of the whole genome [3,11]. Polyproteins 1a and 1b are encoded by ORFs 1a and 1b. These two polyproteins are cleaved with the aid of proteases such as M^pro^ to produce 16 non-structural proteins (NSPs) [9,12,13]. M^pro^, the main protease of the coronaviruses, is responsible for viral replication and transcription and is described in detail in previous studies [12,13]. The NSPs include regulators of the viral lifecycle, which play a crucial role in the replication and pathogenicity of the virus [9,14]. The mechanism of viral invasion and protein functions was reviewed previously [15,16,17]. Downstream in the RNA are 11 accessory proteins encoded by ORFs, whose order is ORF3a, ORF3b, ORF3c, ORF3d, ORF6, ORF7a, ORF7b, ORF8, ORF9b, ORF9c and ORF10, respectively [18]. They interact with four structural proteins, but their roles remain unknown [19]. The four structural proteins are spike surface glycoprotein (S), small envelope protein (E), membrane or matrix protein (M), and nucleocapsid protein (N) [20]. Among the structural proteins, S protein plays a vital role in the pathogenicity and transmission of the virus (Figure 1).

## 3. The Role of Spike Protein in Infection Severity

The spike protein, or S glycoprotein, is a multifunctional homotrimer structural protein of 1273 amino acids and two functional domains, S1 and S2 [23]. The S protein has a primary role in binding the virus into the host cell. It also has a vital role in T cell responses, neutralizing antibody (NAb), and immunity [24]. The spike protein is covered by a glycan shield, which provides flexibility at three points on the stalk and also conceals the surface protein from the immune system [25]. The S1 domain initially mediates the binding of the virus to the host angiotensin-converting enzyme 2 (ACE2) receptors via the receptor binding domain (RBD) [23,26,27,28,29,30]. Previous studies show that amino acids 319–541 of the S1 domain have 10–20-fold higher affinity to human ACE2 in comparison with the RBD of other human coronaviruses [23,31]. The ACE2 binding destabilizes the S1 trimer, so the S2 domain, which comprises a hydrophobic fusion loop, is prepared for membrane fusion and host cell penetration [3,32]. Varieties of host proteases facilitate the cleavage of the spike protein into S1/S2 domains, and each protease has specific characteristics for different coronaviruses [33].

Furin is a primary requisite for the activation of SARS-CoV-2 in human cells by cleaving the S1/S2 domains to prepare the fusogenic envelope [33,34,35,36]. Interestingly, the S1/S2 furin-like cleavage site is not present in SARS-CoV or MERS-CoV. Wu et al. (2021) suggest that the S1/S2 and S2′, proteolytic sites comprise the RXXR motif, which is not activated by furin in MERS-CoV [22,37]. Furin cleavage sites are also characteristic of Marburg and Ebola viruses [21,33]. A major function of furin is to cleave specific sites in the viral polyproteins to produce active proteins [21]. Critically, the expression of the furin protease is not limited to the respiratory system: furin is distributed in the gastrointestinal tract, lung, liver and kidney, which is a reason for the widespread dissemination and cytotoxic behavior of SARS-CoV-2 [38]. In SARS-CoV-2, a significant 12-nucleotide insertion results in an insertion of a 4 amino acid sequence (PRRA), including dibasic residues at positions 682 to 685, the junction of the S1/S2 domain. This position is cleaved by furin, which may facilitate a viral tropism in the host body and make SARS-CoV-2 more pathogenic than previous coronaviruses [38]. Then, transmembrane serine protease 2 (TMPRSS2), a cell surface protease, cleaves the S2′ site at the N-terminal of S2 domain for further exposure of the M protein to the cell membrane, leading to virus–cell fusion [21,22].

Furin plays a key role in distributing the virus and hijacking human cells during COVID-19. The high affinity of the RBD of S protein to the human ACE2, as well as furin activity in many organs, induces wide cell tropism by SARS-CoV-2 and may lead to organ failure in critically ill patients [39]. Furthermore, furin overexpression in diabetes also may increase the probability of severe infection by SARS-CoV-2 [40]. However, other properties of spike protein, such as toxicity, can intensify the pathogenicity of SARS-CoV-2. For example, the spike protein of SARS-CoV-2 can induce coagulation of the platelets in the blood stream of severely infected patients [36]. This may be associated with platelet hyperactivity as an inflammatory response to high levels of SARS-CoV-2 RNA in the blood stream. As well, the spike protein may stimulate platelets along with a hyperinflammatory response, inducing coagulation factors. The possible correlation with elevated fibrinogen and CRP (C-reactive protein) with hypercoagulability may lead to stroke in patients with severe COVID-19 [41]. Thus, the S protein may stimulate arterial thrombosis, which is also the main concern for recipients of the current spike-based vaccines. Therefore, there is a need to find alternative target proteins or methods for vaccine development.

## 4. Why Delta and Lamba Variants Are More Pathogenic

**Figure 2:**
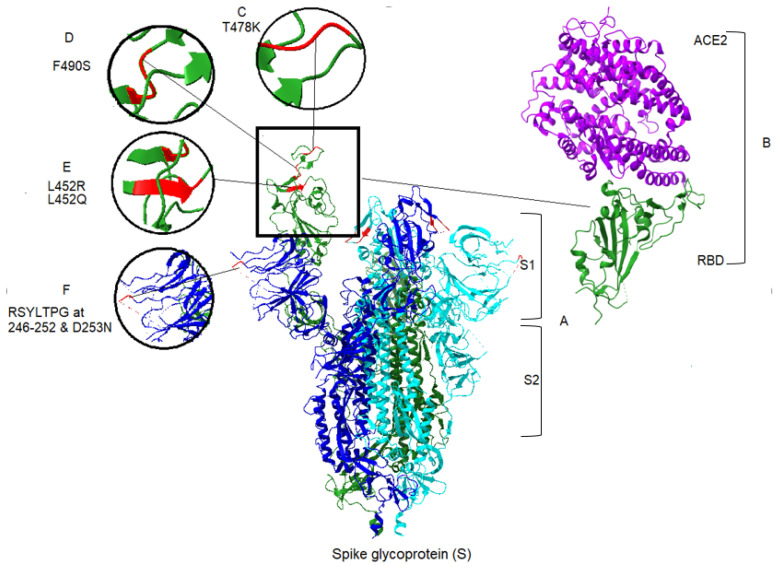
Spike protein and associated mutations in S1 domain for Delta and Lambda variants of SARS-CoV-2, and ACE2 binding to the S protein. (**A**) The crystal structure of S protein domains (S1 and S2) in the active conformation of the RBD of spike protein (Chain A). The PDB format was obtained from the RCSB protein data bank (PDB ID: 7DX5) [49]. Homology modeling was performed, using UCSF ChimeraX version 1.2.5 (2021-05-24). ACE2 was removed from the structure in the central panel [50]. (**B**) Right panel, the partial conformation of the structure of RBD of the spike protein (Chain F), shown in a green color, in complex with ACE2 (Chain B) in purple color, retrieved from the RCSB protein data bank (PDB ID: 6vw1) to demonstrate the RBD binding to hACE2 [51]. Structural modeling and point mutations were carried out, using UCSF ChimeraX. Other chains and molecules were removed. (**C**) The structural comparison of T478K related to the Delta variant. (**D**) The structural demonstration of F490S related to the Lambda variant. (**E**) Demonstration of two mutants related to the two variants, L452R and L452Q of Delta and Lambda, respectively. (**F**) Demonstration of sequence deletion (RSYLTPG at position 246–252) and a mutation D253N located in the NTD of the spike protein of the Lambda variant.

Apart from the toxicity of the S protein, the S1 domain is highly mutable and may not be a suitable candidate for drug targeting [29]. In contrast, cell-mediated immune responses to the S protein are mainly elicited to the S2 domain [42]. This also contains two heptad repeat regions (HR1 and HR2), which provide the S2 domain as a target of NAbs and vaccine candidate [43]. Mutations in spike protein may lead to increased affinity between the RBD and the ACE2 [30,44]. Among the 17 mutations of the Delta spike protein, the L452R and T478K mutations are notable because amino acid substitutions at positions 452 and 478 have increased affinity of the spike protein for ACE2 receptors. Thus, they increase the capability of the virus to evade a host’s immune response [43]. There is a specific mutation, P681R, found within the furin cleavage site of the Delta variant [45]. An initial study in Delta demonstrates the increased processing of spike protein, compared with the original SARS-CoV-2 [46]. In this regard, higher fusion activity and syncytium formation has been reported in the Delta variant, compared with previous lineages [44]. Similarly, there are 19 mutations in the Lambda variant, including a unique deletion of 7 amino acids at positions 246–253, plus the substitution D253N located at the NTD of the S protein (Figure 2), the antigenic supersite, raising the prospect of more transmissibility and more infection in the Lambda variant [8]. However, T76I, F490S and L452Q substitutions in Lambda also occur in the RBD of spike protein [47]. As seen in Figure 2, the most significant changes within the RBD for the two variants are L452R and T478K in Delta, and T76I, F490S and L452Q in Lambda. Generally, mutations that occur in the RBD and the N-terminal domain allow the virus to evade the host immune response. Consequently, the Delta and Lambda variants appears to have decreased sensitivity to antibodies [6,48] elicited by the current vaccine spike protein. The molecular structure, important amino acid changes and protein–protein binding of spike with ACE2 for Delta and Lambda variants are represented in Figure 2.

The genomic data and protein sequence analysis of Delta and Lambda variants were compared with the original Wuhan isolate, available on the NCBI and GISAID clade database, accessed on 29 August 2021 from (http://gisaid.org). The comparison shows abundant changes in NSPs and S proteins (Table 1). The very large number of mutations in Delta and Lambda, combined with the limited experimental data, make the analysis of the significance of mutations extremely difficult at the current time. According to the available sequences of accessory proteins and comparison to the Wuhan strain, ORF6 and ORF7b of Delta and ORFs 6, 7a and 7b of Lambda and ORF10 of both variants had no changes. More information and amino acid sequences are available in Table A1. There are 20 substitutions in Delta’s NSP and 15 substitutions in Lambda’s NSP, plus 2 deletions of trimers. Within the S protein, Delta has 7 amino acid substitutions, while Lambda has 7 amino acid substitutions plus a heptad deletion. The Lambda changes provide replication advantages [52]. The substitutions stabilize the S protein with human ACE2, causing the virus to evade NAbs and increasing COVID-19 severity [6,48,52,53]. There is more conservation in E and M proteins. For Delta and Lambda, there are no changes in the E protein and single amino acid substitutions in the M protein. With regard to the N protein, Delta has 4 amino acid substitutions, while Lambda has 8.

**Figure 3:**
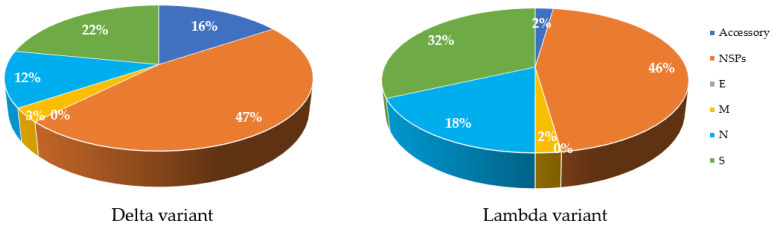
Comparison of amino acid changes in structural and Non-structural proteins in Delta and Lambda variants of SARS-CoV-2 compared to the Wuhan strain. Most changes are observed in NSP proteins (47% for Delta and 46% for Lambda), shown in orange colour in both variants. S proteins, shown in green color, have the greatest changes compared to other structural proteins. E protein, shown in grey color, has no mutations (0%) in both variants. Accessory proteins, shown in dark blue of Lambda show less change (2%) compared to Delta variant (16%). M proteins, shown in yellow color, have the least change in both variants, apart from E protein. The initial databases were extracted from GISAID online tool and processed using Excel.

Mutations are a key factor to determine the characteristics of the virus in terms of severity and infectivity. The number of mutations in structural and non-structural proteins in both Delta and Lambda variants in comparison with the Wuhan isolate are available on GISAID (using CoVsurver: Mutation Analysis of hCoV-19), accessed on 29 August 2021, (https://www.gisaid.org/epiflu-applications/covsurver-mutations-app). In comparison to the original Wuhan strain, the Delta variant has 47% of its total differences in NSPs, while the Lambda variant has 46% (Figure 3). The Lambda variant also has more mutations in S and N proteins (Figure 2). In contrast, the M and E proteins of both variants have incurred one or no change (Figure 3). Analysis of the protein structure changes due to nucleotide sequence changes and determination of how these changes affect protein–protein interactions using bioinformatic tools can lead to new approaches for treatment. Proteins with high sequence conservation, such as E or M, may be a better choice for therapeutic purposes. Further details about the frequency of changes are listed in Table A2.

## 5. Impacts of Changes in New Variants

Current COVID-19 prevention is focused on spike-based vaccines, so the changes in spike protein in Delta and Lambda variants may be considered to affect severity, pathogenicity and transmissibility. A summary of what is known or about the effect of changes in the spike protein in Delta and Lambda variants is shown in Table 2. In instances where data are not yet available, we speculate on the effects.

The Delta variant with the L452R substitution showed less sensitivity to NAbs [6,48]. The ACE2 binding to the S protein in the Delta variant exposes the furin cleavage site and increases the S1/S2 cleavage rate by furin [6]. Consequently, the replication of the Delta variant in the human airway is higher than previous variants [44]. There are two amino acid changes: L452R and T478K. Arginine is hydrophilic and polar in nature, and it can accelerate molecular transmission and infectivity in the mucosal environment [53,54,55,56]. However, the mechanism of interaction of arginine within the membrane is unknown. A recent study showed that mutation in position L452 frequently escapes neutralizing antibodies [57]. This position demonstrated two mutations, including L452R and L452Q, in the Delta and Lambda variants, respectively. Due to the T478K change, an uncharged amino acid threonine is replaced with a positively charged and basic lysine. This amino acid replacement leads to increasing the electrostatic interaction in the spike protein, enhancing transmission and the protein–protein binding affinity of the virus to ACE2 in an extracellular environment [58]. One study reported that the larger side chain of lysine can impact the steric structure of the Delta variant and ultimately may enhance the interaction of the spike protein to ACE2 [59]. The change L452Q occurs in the Lambda variant. The amino acid glutamine is polar and contains a neutral side chain. However, this change represents the same property as L452R in the Delta variant and similarly causes increased affinity to ACE2 [60]. The mutation F490S occurs in the Lambda variant. Phenylalanine is a very hydrophobic amino acid, while serine is neutral; it is expected that the charge change increases viral infectivity [60]. Further information related to amino acids that affect SARS-CoV-2 infection is described elsewhere [54]. The Lambda mutations that contribute to increased viral infectivity and resistance to vaccine elicited serum include T76I, F690S and L452Q [8,60]. However, the major concern about Lambda prevalence is associated with the deletion of key sequences, ^246^RSYLTPG^252^, and substitution D253N. The current vaccines target these sequences to protect individuals. The deletion of these key sequences leads to reduced vaccine efficacy against Lambda infection in vaccinated individuals [8,60].

## 6. Potential Target Proteins for Vaccine Development

The polymorphism of the spike protein may impact the effectiveness of current vaccines focused on the spike, so it is desirable to investigate alternative proteins as therapeutic targets [35]. The spike protein can directly cause platelets to release a coagulation factor, leading to the secretion of inflammatory cytokines and fatal results [36,61]. There were higher death rates among patients with previous health conditions, such as individuals with diabetes and cancer, as well as the elderly [41,62,63]. In elderly people with illness and reduced homeostasis, protein clearance is poor, resulting in the risk of severe infections [64].

Some evidence suggests that accessory proteins may interfere in the pathogenicity and transmissibility of the virus [65]. For example, an experimental study identified the ORF3a protein as a potential protein in viral pathogenicity [62]. Excess mutations occurring in ORF1a, ORF8, and NTD provide other instances of missense mutations within the accessory proteins, which alter the function of the protein in favor of increasing the infectivity and transmissibility of the virus [63]. In contrast, the accessory proteins demonstrate less variation and might be potential alternatives for vaccine development [3]. For instance, E protein and ORF6 and ORF7b proteins demonstrate less mutation and might be suitable therapeutic candidates, although several hurdles need to be overcome to determine the significance of parts of the E protein [66]. ORF10 is also suggested to encode a functional protein and may be considered a target for vaccine development [67,68].

N protein is another alternative that has demonstrated more stability, compared with the spike protein of SARS-CoV-2 [26]. Despite the N protein demonstrating more changes than the spike protein in other coronaviruses, N shows more conservation in SARS-CoV-2 [69]. Within N protein, epitopes have been identified in association with B- and T cells, and these epitopes have remained conserved in Delta and Lambda variants [69]. Therefore, targeting these epitopes in N protein might provide immune protection against the SARS-CoV-2 variants [69]. Among the detected epitopes of N protein in convalescents, the ^404^SKQLQQSMSSADS^416^ and ^92^RRIRGGDGKMKDL^104^ epitopes were identified for B cell receptors [26,69]. Salvatori et al. (2021) suggest a new mechanism of vaccination based on the antibodies produced against the N protein [70]. The number of antibodies against N protein produced during the infection by SARS-CoV-2 are greater than the antibodies against S protein [70]. The N protein is located inside the virus and wraps the genome of the virus: understanding the mechanism of neutralization of the N protein requires further investigation. Since most research focuses on blocking viral entry rather than triggering viral particle assembly, the surface glycoprotein has been the major target of NAbs [70]. However, some antibodies work inside the cell and Foss et al. (2012) show that TRIM21 is a high-affinity antibody receptor inside the cell, facilitating N protein recognition by antibodies inside the cell [71]. The N protein antibodies bind the N protein inside the cell and cleave it so that fragments of N protein reach the cell surface, where the T cells recognize the infected cell [71].

This review shows that there are a few potential therapeutic pathways other than the current vaccine production methods that require more focus to find alternative treatments and vaccines.

## 7. Conclusions

The high pathogenicity and rapid transmission of SARS-CoV-2 have led the world to the current pandemic. Changes to the behavior of the virus by frequent mutation cause challenges for vaccine development. The S protein is one of the most mutable parts of SARS-CoV-2: changes to the S protein can facilitate more transmission and evasion of NAbs, specifically in the Delta and Lambda variants. It seems that higher affinity of the S1 domain of the S protein to the ACE2 is one of the main reasons for the higher pathogenicity of the Delta and Lambda variants [44,47]. Toxicity of the S protein to stimulate thrombosis is the notable concern in current mRNA and adenovirus vaccines which express the S protein. Toxicity of the S protein can result in stimulation of platelet activity and the release of coagulation factors, which may cause thrombosis in infected individuals. Due to high mutation in the S protein, which may cause immune escape in new variants and a toxicity property, the investigation of alternative proteins or treatment other than spike-based vaccines to stimulate an immune response is suggested.

Targeting non-structural proteins and accessory proteins, particularly ORF10, could be another option to prevent infection and to stimulate treatment [68]. The redundant cleavage site of furin in the S protein may be another reason for the high pathogenicity in SARS-CoV-2. A redundant furin cleavage provides rapid viral replication in many organs, tissues and especially the respiratory tract of the infected individuals, leading to increased viral load. The presence of a furin cleavage site in the S protein is novel in betacoronavirus [72]. Notably, there may be a correlation between the substitution within the furin cleavage site in Delta and increased transmission in this variant. Therefore, it is hypothesized that the action of furin increases the infectivity of SARS-CoV-2 in infected individuals without chronic disease. This hypothesis needs further investigation.

The changes in the new variants may be associated with the increased hydrophilicity and charge of the S protein, which may lead to higher transmissibility of the virus. However, this hypothesis also requires further research to determine the role of amino acid changes in the pathogenicity of SARS-CoV-2. Despite the Lambda variant demonstrating multiple changes and a sequence deletion, and its potential to become a VOC, there are limited data about this variant. Therefore, with limited access to metadata, it is challenging to predict whether Lambda can escape NAbs in vaccinated individuals in the future. These data warrant further investigation of the Lambda variant.

Finally, some mutations in new variants could increase the S protein affinity to ACE2 and furin and enhance the extracellular transmission. Furthermore, amino acids deletions and changes in antigenicity could diminish immune responses in vaccinated individuals. Hence, it may be better to design a vaccine with a combination of S and N proteins to increase the efficiency of the immune response.

It should be noted that this study had limitations in terms of investigation on epitope specificity of SARS-CoV-2, to determine which epitopes can be considered as molecular targets for neutralizing antibodies. A recent study identified more than 1400 epitopes associated with human T cells [72]. Further studies are needed to determine the important epitopes of the Delta and Lambda variants.

## Figures and Tables

**Table 1:** List of amino acid (aa) substitutions and deletions of Delta and Lambda variants in comparison with the original Wuhan strain. The non-structural proteins (NSPs) and accessory proteins in beige, envelope protein (E) in cyan, membrane protein (M) in green, nucleocapsid protein (N) in yellow, and spike glycoprotein (S) in dark orange color represent different changes for both Delta and Lambda variants of SARS-CoV-2, using GISAID ID: EPI_ISL_402124. The Lambda variant has more changes, compared to the Delta variant, and shows a deleted amino acid sequence in NSPs and S proteins. Delta and Lambda sequences extracted from NCBI tool and changes were found, using the GISAID online tool. Changes in accessory proteins ORF3, ORF7a and ORF8 in Delta, and ORF8 in Lambda are highlighted in dark blue color.

COVID-19 Variant	aa Substitutions Deletions						Source
	NSPs Accessory Proteins		E	M	N	S	
**Delta (δ)**	**NSP3:**	P822L, T1022X, P2767L, T2967X	No Change	I82T	D63G, R203M, Q349H, D377Y	T19R, A222V, L452R, T478K, D614G, P681R, D950N	NCBI, GISAID (EPI_ISL_402124)
	NSP4:	A446V, A946V					
	NSP6:	V149A, T181I, V439A, T471I					
	NSP12:	P323L, M463I, G671S					
	NSP13:	P77L, T125N					
	**ORF3:**	**S26L**					
	**ORF7a:**	**V82A, T120I**					
	**ORF8:**	**D119V, F120L**					
**Lambda (λ)**	NSP3:	T428I, P1469S, F1569V, T2373I, P3414S, F3514V	No Change	K384N	P13L, R203K, G204R, G214C, P432L, R622K, G623R, G633C	G75V, T76I, R246Δ, S247Δ, Y248Δ, L249Δ, T250Δ, P251Δ, G252Δ, D253N, L452Q, F490S, D614G, T859N	NCBI, GISAID (EPI_ISL_402124)
	NSP4:	L438P, T492I, L938P, T992I					
	NSP5:	G15S, G321S					
	NSP6:	S106Δ, G107Δ, F108Δ, S396Δ, G397Δ, F398Δ					
	NSP12:	P323L					
	NSP13:	T599I					
	**ORF8:**	**S142N**					

**Table 2:** Impact of the substitution and deletion of amino acids in spike protein on pathogenicity of Delta and Lambda variants. The Delta substitutions in S protein are shown, and the effectiveness of each change is indicated. Amino acids substitutions of the Lambda are represented. The Lambda variant indicates a novel heptad amino acids 246–252 deletion plus a D253N substitution in the S protein with higher impact on pathogenicity. The impact of each mutation is addressed.

Variant	aa Substitution or Deletion	Impact of Mutation on Pathogenicity	Reference
**Delta (δ)**	T19R	Also found in some Alpha variants/located in NTD supersite and targeted by most anti-NTD neutralising antibodies	58
	A222V	Conservative change	59
	**L452R**	**Located in RBD and increase affinity to ACE2/ impairs neutralisation by antibodies/Increases transmissibility **	6, 49, 58
	**T478K**	**Located in RBD/increases affinity to ACE2/increases virus transmissibility**	6, 49, 58
	D614G	Also found in some Alpha, Gamma and Lambda variants	55, 57
	**P681R**	**Located in the furin-cleavage site/increases the fusogenic activity of the spike protein**	62, 49, 47
	D950N	Unknown	_
**Lambda (λ)**	G75V	Conservative change/not effect on pathogenicity	8
	**T76I**	**Increases viral infectivity/causes partial resistance to vaccine**	62
	**Deletion of RSYLTPG at position 246–252**	**Confers partial resistance to vaccine immunity**	8, 45, 49, 62, 65
	**D253N**	**Confers partial resistance to vaccine immunity**	8, 45, 49, 62, 65
	**L452Q**	**Contributes transmissibility/causes partial resistance to vaccine elicited serum**	62
	**F490S**	**Novel mutation position in RBD/contributes to transmissibility/causes partial resistance to vaccine elicited serum**	62
	D614G	Also found in some Alpha, Gamma and Delta variants	62
	T859N	Found in the Beta variant/associated with decreased neutralization by monoclonal antibodies	48

## Data Availability

Not applicable.

## Appendix A

**Table A1:** Accession number of protein sequence of SARS-CoV-2, Delta and Lambda variants available on NCBI, compared with the original Wuhan strain available on GISAID to identify substitution and change in amino acid sequence in both new variants.

SARS-CoV-2 Proteins	Accession Number	
	Delta Variant	Lambda Variant
ORF10	UAL04656.1	QXG22373.1
N protein	UAL04655.1	QXG22372.1
ORF8	UAL04654.1	QXG22371.1
ORF7b	UAL04653.1	QXG22370.1
ORF7a	UAL04652.1	QXG22369.1
ORF6	UAL04651.1	QXG22368.1
Membrane glycoprotein	UAL04650.1	QXG22367.1
E protein	UAL04649.1	QXG22366.1
ORF3a	UAL04648.1	QXG22365.1
S protein	UAL04647.1	QXG22364.1
ORF1a	UAL04646.1	QXG22363.1
ORF1ab	UAL04645.1	QXG22362.1

**Table A2:** Percentage of the amino acid change in structural and non-structural proteins in both Delta and Lambda variants of COVID-19, compared to the Wuhan strain.

COVID-19 Variant	aa Substitution						Total
	Accessories	NSPs	E	M	N	S	
Delta	5.00	15.00	0.00	1.00	4.00	7.00	27.00
%	15.63	46.88	0.00	3.13	12.50	21.88	
Lambda	1.00	20.00	0.00	1.00	8.00	14.00	43.00
%	2.27	45.45	0.00	2.27	18.18	31.82	
